# Interrelationship between arterial hypertension, health service costs, therapeutic treatment and physical activity

**DOI:** 10.1186/s12875-023-02120-7

**Published:** 2023-08-25

**Authors:** Lionai Lima dos Santos, Jamile Sanches Codogno, Bruna Camilo Turi-Lynch, Monique Yndawe Castanho Araujo, Romulo Araujo Fernandes, Grace Angelica de Oliveira Gomes, Shirley Crankson, Nana Anokye

**Affiliations:** 1 Department of Physical Education, São Paulo State University - UNESP, Presidente Prudente, São Paulo, Brazil; 2 Department of Physical Education and Exercise Science, Lander University, 320 Stanley Avenue, 29649 Greenwood, SC USA; 3 Department of Gerontology, Federal University of São Carlos, São Carlos, São Paulo, Brazil; 4 Department of Clinical Sciences, College of Health and Life Sciences, Brunel University London, London, UK

**Keywords:** Cardiovascular disease, Health expenditures, Physical exercise, Treatment adherence

## Abstract

**Background:**

Arterial hypertension is a high prevalence disease that increase healthcare costs and affects physical activity level. This study aimed to analyse the interrelationship between arterial hypertension, health service costs, therapeutic treatment, and physical activity in patients with cardiovascular diseases.

**Method:**

Cross-sectional study that evaluated 306 patients from a hospital in Presidente Prudente-Brazil. Based on their medical diagnosis, they were classified into multiple groups to access primary care and hospital-related costs variations. Then, using data from medical records and face to face interviews, they were examined on their treatment adherence and physical activity practice. Healthcare costs were accessed using medical records. Finally, the generalised linear model was used to analyse the interrelationship between treatment adherence, physical activity, health care costs and arterial hypertension. The data were analysed with Stata/MP4 16, and a p-value of less than 5% was used to determine statistical significance.

**Results:**

The group that adhered to the arterial hypertension treatments but were physically inactive presented higher costs with consultation (US$=24.1, 95%CI = 1.90;46,3)​​ medication (US$=56.60, 95%CI = 1.65; 111.5) and total primary health care costs (US$=71.60, 95%CI = 19.2; 123.9) even after adjusting for confounding variables, meanwhile those participants that adhered to the treatments and were physical active did not present difference in healthcare cost when compared to normotensive and physical active participants.

**Conclusion:**

To be adherent to hypertension treatment were related to higher health care costs meanwhile been physical activity were related to lower health care costs and the combination of both showed that be adherent and physical activity represent the same cost with health than those normotensive and active emphasizing the importance of adherence and physical activity in the hypertensive treatment.

## Background

Arterial hypertension (AH) is the most prevalent cardiovascular disease in the world. Surveys indicate that in 2015 about 1.13 billion people had AH [1]. A study with 382,341 people from five continents, aged between 35 and 70 years old, showed a prevalence of AH of 40.8%, whereas in people over 60 years old, it showed a prevalence above 60% [2]. In the United States, AH prevalence of 34.9% [3] was reported. Also, in Brazil, the prevalence of AH was 32.5% in adults under 60 years old and 60% in adults over 60 years old [4]. A high prevalence of AH is associated with significant economic impact, as AH requires continuous treatment [5], leading to an increase in health care costs [6]. In the United States, in 2014, an estimate of US$ 126.7 billion accounted for AH treatment [7]. In Europe (Italy, Germany, Spain, France and England), the total costs of cardiovascular events attributed to AH in 10 years were valued at € 51.29 billion [8], while in Brazil, the total costs of medical assistance due to AH in the public health system was estimated at approximately US$ 400 million in 2005 [9].

Current literature provides evidence-based treatments that help control AH. These treatments help reduce cardiovascular events and, consequently, reduce costs with health services [10, 11]. Apart from lowering health care costs, the benefit of AH treatment adherence has been associated with reduced mortality and morbidity. For example, LU et al. (2022) [12] found that participants who used antihypertensive medication and had a favourable lifestyle presented lower risk of all-cause, cardiovascular and cancer mortality compared to participants not using antihypertensive medication and following an unfavourable lifestyle. Also, another study observed that AH treatment adherence could decrease cerebral vascular events by 30 to 45% [13]. Although treatment adherence is a big challenge to most populations, its minimised health care costs are beneficial. For instance, a study showed that a 70% increase in AH treatment adherence in the countries studied (Italy, Germany, Spain, France and England) could reduce service costs by €332 million in 10 years [8].

Furthermore, other studies have shown that physical activity is effective non-medication management of AH. Consequently, it could reduce health care costs for physically active AH patients. For example, the literature shows that the practice of moderate physical activity can keep blood pressure values ​​low until 24 hours after the practice [6]. Furthermore, Bueno et al. (2017) [14] showed that physically active patients have drug cost savings of 395% compared to physically inactive patients. Additionally, Turi and collaborators (2017) [15] noted that a person with AH who is physically active could have a US$ 50 reduction in their primary health care costs per year compared to the physically inactive group.

Our literature review identified limited evidence, in national and international level, of studies that considered in the same analysis the influence of AH, treatment adherence and physical activity on health service cost. Therefore, the present study is justified by the need for studies that elucidate these relations. It should be noted that the present methodology involves direct extraction of information from medical records and analysis of data related to expenses and outcomes in public health, which, in addition to being easy to apply and low cost, are of great value not only for public policy strategies but also for health professionals involved in the prevention, control and treatment of arterial hypertension.

Thus, the objective of this study was to analyse the interrelationship between AH, health service costs, treatment adherence and habitual physical activity through robust data analysis activity.

## Methods

### Sample selection and ethical procedures

Cross-sectional study that was conducted in Presidente Prudente (~ 200,000 inhabitants, human development index of 0.806) in the western region of Sao Paulo State, Brazil. The data of 306 selected participants were retrospectively accessed and included in this study. For this study, we included only hospital patients within the ICD-10 International Classification of Diseases and Health-Related Problems, specifically patients who had cardiovascular diseases that fell within the ICD-I category. In addition to the data mentioned above, the inclusion criteria for the research were: i) patients aged between 30 to 65 years old [16]; ii) at least one active health care attendance in the Public Health System in the last 12 months; iii) regular/permanent residence in Presidente Prudente. These last criteria was used due to the authorisation of the Health Department of Presidente Prudente to obtain information about primary health care services.

The annual number of patients seen at the Presidente Prudente hospital (163,288 visits) informed this study’s minimum sample size calculation. Then, based on the number of visits, we calculated the percentage of people in the city of Presidente Prudente aged between 30 and 65 years and fell within the ICD-I category (~ 0.74% of all visits; 1,200 visits). Thus, with 0.74% visits, a sample error of 5% and Z = 1.96, a minimum sample size of 178 people was considered. However, 306 patients selected at random were included in this study.

The eligible patients were contacted by telephone and invited to participate in a face-to-face evaluation and interview. Those who agreed to participate signed a consent form. The study was approved by the Research Ethics Committee of the Sao Paulo State University, Presidente Prudente Campus (CAAE 82767417.5.0000.5402).

### Protocol

The first stage of the research focused on obtaining the necessary authorisations to carry out the research. Initially, contact was made with the hospital and authorisation was requested to carry out the study, including access to patient data. After, the hospital’s authorisation the protocol was submitted to the Research Ethics Committee.

After Research Ethics Committee approval, patients who visited cardiologists in the last 12 months at the hospital and met this study’s eligibility criteria were accessed. From this list and using specific software, randomization of the patients was carried out, the patients drawn were telephone invited to participate in the research.

The patients who agreed to participate in this research were invited to a face-to-face interview that assessed their physical activity participation using a questionnaire. After the face-to-face assessments, their medical records were accessed through the Secretary of Health (primary care) and the hospital (secondary and tertiary care) information system. Finally, their data from the face-to-face interviews and medical records were included in the data analysis.

### Dependent variables

#### Hospital care costs - secondary and tertiary care costs

The total hospital-related expenditure was estimated from both the secondary and tertiary care costs of the patients. This data was accessed from Presidente Prudente hospital records. It included information on spending from emergency room consultations, scan and laboratory tests, hospitalisations, surgeries, blood bags, human resources, length of hospital stay, medical procedures and used resources, medicines and consumables.

#### Primary care costs

For primary health service costs, the information recorded in the electronic database of the city’s health department was used, including the 12 months before the patient’s inclusion in this study. The use of health services and the monetary values ​​of each expenditure were estimated as follows:

- Medical consultations and other professional consultations (social work, nursing, pharmacy, physical therapy, speech therapy, nutrition, dentistry, psychology, occupational therapy). The information provided by the ministry of health was used to calculate the costs of each of these consultations.
- Medications expenditure and laboratory tests. The service costs were calculated through the specific cost of each service multiplied by the number of procedures performed by each patient.

The total costs referring to the expenses of primary health care still included:

- Patient care services (scheduling appointments, medication dispensing, management, among others). We considered the daily salary of the professionals involved, the value of daily work (total pay divided by 30 days) and the average number of patients attended daily to calculate the costs of each service.
- Utility bills (electricity, water, and telephone). We used the average values of the last three months, then the daily value of each bill (average value divided by 30 days), and the unit value corresponding to each utility bill expenditure for each participant (daily value divided by the number of patients treated daily), according to the methodology used in previous studies [17–19] to calculate costs with utility bills.

Later, the costs evaluated in Brazilian Reais, was converted into dollars according to the Central Bank of Brazil dollar rate on October 1, 2018 (1 US$ = 4.0267BRL), and corrected by dollar inflation rate (1.62%) from October 2018 to December 2019. The conversion rate was obtained from the official Government website (https://data.bls.gov/cgi-bin/cpicalc.pl).

### Independent variable

#### Arterial Hypertension (AH)

The presence of AH was self-reported at the interview and confirmed through medical records. Additionally, blood pressure measurements were also performed following the protocol of the VII Brazilian Guideline for Arterial Hypertension [6].

#### Treatment adherence

Adherence to treatment among patients was assessed with the Martín-Bayarre-Grade Questionnaire (MBG) [20]. This tool is validated for the Brazilian context [21]. The questionnaire has 12 items in the form of affirmations, with five options of Likert scale answers ranging from “never” to “always” (from 0 to 4 points). The final score is categorised into three groups: non-adherent (0 to 17 points), partially adherent (18 to 37 points), and adherent (38 to 48 points)18. For statistical analysis, two groups were composed: non/partially-adherent (0–37 points) and adherent (38–48 points).

#### Habitual physical activity

The Habitual Physical Activity (HPA) of the patients was assessed with the questionnaire developed by Baecke et al. (1982) [22] and translated and validated by Florindo et al. (2004) [23]. The questionnaire assessed the level of HPA in three domains: occupational physical activities, physical exercises practised at leisure time, and leisure and locomotion physical activities [22, 23]. For statistical analysis, the usual physical activity score was divided into quartiles; patients classified in the highest quartile (P ≥ 75) were considered physically active, and those in the lowest quartile (p < 75) were considered insufficiently active.

Considering the independent variables of the study, for statistical analyses, patients were divided as follows according to HPA classification and AH diagnosis (Tables 1 and 2). The groups were: i) normotensive + physically active (NOR + ACTIVE) [reference]; ii) normotensive + insufficiently active (NOR + IA); iii) hypertensive + physically active (AH + ACTIVE); iv) hypertensive + insufficiently active (AH + IA).

**Table 1:** Descriptive variables according to presence of arterial hypertension. adherence to treatment and level of physical activity

	NOR+ACTIVE (n = 30)	NOR + IA (n = 72)	AH_NOTADH_+ACTIVE (n = 137)	AH_NOTADH_+IA (n = 43)	AH_ADH_+IA (n = 16)	AH_ADH_+ACTIVE (n = 8)	p-value
	Median (IR)	Median (IR)	Median (IR)	Median (IR)	Median (IR)	Median (IR)	
Age	48.3 (11.7)	52.5 (12.5)	58.9 (10.6)^a. b^	54.9 (12.1)^a. c^	56.9 (10.6)^a^	54.0 (10.6)	**0.001**
BMI	27.4 (6.9)	28.4 (6.8)	31.0 (7.4)^a. b^	31.3 (6.5)^a. b^	29.1 (7.1)	28.9 (6.0)	**0.001**
Weight	72.4 (14.8)	79.7 (24.3)	81.3 (16.7)^a^	81.7 (18.7)^a^	77.2 (24.4)	78.4 (27.5)	**0.014**
WC	90.3 (17.6)	97.8 (16.5)	103.0 (15.8)^a. b^	103.7 (14.5)^a. b^	95.3 (18.1)	98.9 (17.8)^c. d^	**0.001**
SBP	120.0 (30)	110.0 (20)	125.0 (30)^a. b^	130.0 (40)^a. b. c^	125.0 (28)^b^	135.0 (18)^a. b^	**0.001**
DBP	80 (20)	80 (20)	80 (10)^b^	90 (20)^a. b^	80 (18)	80 (10)	**0.004**
Sum of diseases	0.00 (0.2)	0.00 (1.0)	2.0 (2.0)^a. b^	2.0 (2.0)^a. b^	2.0 (2.0)^a. b^	3.0 (2.7)^a. b^	**0.001**
**Costs**							
Consultation	17.4 (22.2)	17.5 (19.5)	32.9 (36.9)^a. b^	26.05 (39.0)^a. b^	39.8 (46.2)^a. b^	42.0 (32.5)^a. b^	**0.001**
Laboratory tests	0 (6.1)	0 (11.5)	0 (16.0)^a^	7.22 (21.7)^a^	3.54 (13.2)	0 (5.6)^d^	**0.029**
Medication	9.69 (28.4)	2.27 (11.2)^a^	43.4 (64.7)^a. b^	14.9 (46.8)^a. b. c^	68.10 (54.2)^a. b. d^	11.4 (19.5)^b. c. e^	**0.001**
Total primary	42.8 (61.8)	23.5 (42.1)	95.7 (85.9)^a. b^	54.7 (104.6)^a. b. c^	121.2 (123.3)^a. b. d^	58.8 (43.8)^c. e^	**0.001**
Total Sec. e ter.	264.6 (422.9)	219.5 (378.3)	363.3 (774.8)^a. b^	236.3 (738.4)	368.3 (1241.5)	345.7 (1094.5)	0.101

Notes: p-value < 5%. Kruskal Wallis used to check the difference for more than three Mann-Whitney groups used to check the difference between two groups. NOR + ACTIVE = normotensive + physically active. NOR + IA = normotensive + insufficiently active. AH_NOTADH_+ACTIVE = hypertensive not/partially adherent + physically active. AH_NOTADH_+IA = hypertensive not/partially adherent + insufficiently active. AH_ADH_+ACTIVE = hypertensive adherent + physically active. AH_ADH_+IA = active hypertensive adherent + insufficiently active. ^a^ = difference in NOR + ACTIVE. ^b^ = difference in NOR + IA. ^c^ = difference in AH_NOTADH_+ACTIVE. ^d^ = difference in AH_NOTADH_+IA. ^e^ = difference in AH_ADH_+IA. IR = interquartile range. BMI = body mass index. WC = waist circumference. SBP = systolic blood pressure. DBP = diastolic blood pressure. sec = secondary. ter = tertiary

**Table 2:** Marginal effect expenses estimates in patients with arterial hypertension according level physical active in a crude analyse.

	Marginal effects (US$)									
	Consultation		Laboratory tests		Medication		Total primary care		Total sec. and terc. care	
	Estim. (S.E.)	[95% CI]	Estim. (S.E.)	[95% CI]	Estim. (S.E.)	[95% CI]	Estim. (S.E.)	[95% CI]	Estim. (S.E.)	[95% CI]
NOR + ACTIVE (reference) (n = 31)	1.0	1.0	1.0	1.0	1.0	1.0	1.0	1.0	1.0	1.0
NOR + IA (n = 69)	5.9 (4.4)	-2.7; 14.6	-2.8 (2.2)	-7.2; 1.5	8.2 (4.9)	-1.4; 17.9	11.3 (8.0)	-4.4; 27.1	19.7 (178.9)	-330.8; 370.4
AH + ACTIVE (n = 51)	17.5** (6.0)	5.6; 29.3	2.4 (2.9)	-3.3; 8.2	20.0** (7.5)	5.2; 34.8	40.0** (11.4)	17.5; 62.4	155.6 (209.5)	-255.1; 566.4
AH + IA (n = 156)	23.5** (4.7)	14..3; 32.8	0.4 (2.2)	-4.0; 4.9	43.0** (7.4)	28.4; 57.5	67.0** (9.6)	48.1; 85.9	289.0 (179.1)	-62.1; 640.2

Test GLM. * <5%. ** <1%. S.E = std. error. CI = confidence interval. Estim.= estimate. sec.= secondary. terc.= tertiary. NOR + ACTIVE = normotensive + physically active. NOR + IA = normotensive + insufficiently active. AH + ACTIVE = hypertensive + physically active. AH + IA = hypertensive + insufficiently active.

Subsequently, the patients were divided, considering adherence to the treatment of hypertension (Tables 3, 4 and 5). The groups were: i) normotensive + physically active (NOR + ACTIVE) [reference]; ii) normotensive + insufficiently active (NOR + IA); iii) hypertensive not/partially adherent + physically active (AH_NOTADH_+ACTIVE); iv) hypertensive not/partially adherent + insufficiently active (AH_NOTADH_+IA); v) hypertensive adherent + physically active (AH_ADH_+ACTIVE); vi) active hypertensive adherent + insufficiently active (AH_ADH_+IA).

**Table 3:** Marginal effect expenses estimates in patients with arterial hypertension according level physical active in a adjusted analyse.

		Marginal effects (US$)															
		Consultation (adjusted)			Laboratory tests (adjusted)				Medication (adjusted)				Total primary care (adjusted)			Total sec. and terc. care (adjusted)	
		Estim. (S.E.)		[95% CI]	Estim. (S.E.)		[95%ICI]		Estim. (S.E)		[95% CI]		Estim. (S.E.)		[95% CI]	Estim. (S.E.)	[95% CI]
NOR + ACTIVE (reference) (n = 31)		1.0		1.0	1.0		1.0		1.0		1.0		1.0		1.0	1.0	1.0
NOR + IA (n = 69)		6.48 (4.76)		-2.85; 15.8	-2.83 (2.52)		-7.79; 2.12		12.23 (7.08)		-1.65; 26.12		12.6 (9.70)		-6.38; 31.6	-1.16 (205.2)	-403.4; 401.1
AH + ACTIVE (n = 51)		13.4* (6.15)		1.34; 25.4	2.47 (3.48)		-4.35; 9.30		10.8 (7.83)		-4.53; 26.1		23.7 (1.1)		-0.10; 47.5	121.6 (247.3)	-363.0; 606.3
AH + IA (n = 156)		20.0** (5.20)		9.90; 30.2	-0.08 (2.69)		-5.36; 5.18		34.2** (7.82)		18.9; 49.5		51.6** (10.8)		30.2; 72.9	254.1 (255.7)	-188.4; 696.6
**Confounding variables**																	
Gender (female)		11.6** (4.20)		3.36; 19.8	1.39 (1.46)		-1.47; 4.26		-5.59 (6.93)		-19.1; 7.99		10.9 (8.49)		-5.71; 27.6	-163.8 (130.7)	-420.1; 92.4
Age		0.27 (0.24)		-0.21; 0.75	0.11 (0.91)		-0.06; 0.29		0.40 (0.44)		-0.47; 1.28		0.67 (0.52)		-0.35; 1.71	1.62 (8.02)	-14.1; 17.3
WC		-0.01 (0.13)		-0.27; 0.23	-0.02 (0.04)		-0.12; 0.06		0.10 (0.27)		-0.43; 0.64		0.06 (0.30)		-0.53; 0.65	-6.03 (4.90)	-15.6; 3.57
Sum of diseases		12.7* (5.29)		2.37; 23.1	1.28 (1.77)		-2.18; 4.76		25.8** (10.0)		6.17; 45.6		38.1** (11.4)		15.7; 60.5	128.8 (168.2)	-200.8; 458.4

Test GLM. * <5%. ** <1%. S.E. = std. error. Estim.= estimate. WC = waist circumference. CI = confidence interval. sec.= secondary; terc.= tertiary. NOR + ACTIVE = normotensive + physically active. NOR + IA = normotensive + insufficiently active. AH + ACTIVE = hypertensive + physically active. AH + IA = hypertensive + insufficiently active

**Table 4:** Marginal effect estimates of health care expenditures in patients with arterial hypertension according to adherence to treatment and level of physical activity in a crude analyse

	Marginal effects (US$)									
	Consultation		Laboratory tests		Medication		Total primary care		Total sec. and terc. care	
	Estim. (S.E.)	[95% CI]	Estim. (S.E.)	[95% CI]	Estim. (S.E.)	[95% CI]	Estim. (S.E.)	[95% CI]	Estim. (S.E.)	[95% CI]
NOR + ACTIVE (reference) (n = 30)	1.0	1.0	1.0	1.0	1.0	1.0	1.0	1.0	1.0	1.0
NOR + IA (n = 72)	6.0 (4.3)	-2.5; 14.6	-2.6 (2.2)	-7.0; 1.6	7.5 (4.7)	-1..8; 16.9	10.8 (7.8)	-4.5; 26.3	-5.7 (181.9)	-362.3; 350.7
AH_NOTADH_+ACTIVE (n = 137)	24.0** (4.9)	14.3; 14.3	.8 (2.3)	-3.6; 5.4	42.9** (7.7)	27.7 58.2	67.8** (10.0)	48.2; 87.4	287.4 (189.4)	-83.8; 658.7
AH_NOTADH_+IA (n = 43)	18.0** (6.4)	5.3; 30.6	4.0 (3.3)	-2.5; 10.6	23.3** (8.7)	6.2; 40.5	45.4** (12.7)	20.5; 70.4	156.1 (226.2)	-287.3; 599.6
AH_ADH_+IA (n = 16)	28.0** (12.0)	4.4; 51.7	− .8 (3.2)	-7.1; 5.4	51.0** (23.8)	4.3; 97.8	78.3** (26.3)	26.7; 129.9	268.0 (352.8)	-423.5; 959.7
AH_ADH_+ACTIVE (n = 8)	17.1 (12.9)	-8.2; 42.4	-5.3 (2.2)*	-9.8; -0.8	1.3 (7.8)	-14.0; 16.7	13.0 (16.9)	-20.1; 46.3	62.5 (366.1)	-655.1; 780.1

Test GLM. * <5%. ** <1%. S.E. = std. error. CI = confidence interval. Estim.= estimate. sec.= secondary; terc.= tertiary. NOR + ACTIVE = normotensive + physically active. NOR + IA = normotensive + insufficiently active. AH_NOTADH_+ACTIVE = hypertensive not/partially adherent + physically active. AH_NOTADH_+IA = hypertensive not/partially adherent + insufficiently active. AH_ADH_+ACTIVE = hypertensive adherent + physically active. AH_ADH_+IA = active hypertensive adherent + insufficiently active.

**Table 5:** Marginal effect estimates of health care expenditures in patients with arterial hypertension according to adherence to treatment and level of physical activity in an adjusted analyse.

				Marginal effects (US$)																					
				Consultation (adjusted)					Laboratory tests (adjusted)					Medication (adjusted)					Total primary care(adjusted)			Total sec. and terc. care (adjusted)			
				Estim. (S.E.)		[95% CI]			Estim. (S.E.)		[95% CI]			Estim. (S.E.)		[95% CI]			Estim. (S.E.)	[95% CI]		Estim. (S.E.)	[95% CI]		
NOR + ACTIVE (reference) (n = 30)				1.0		1.0			1.0		1.0			1.0		1.0			1.0	1.0		1.0	1.0		
NOR + IA (n = 72)				6.42 (4.69)		-2.76; 1..6			-2.35 (2.38)		-7.03; 2.32			11.2 (6.96)		-2.40; 24.9			11.9 (9.50)	-6.64; 30.6		-22.5 (208.1)	-430.4; 385.3		
AH_NOTADH_+ACTIVE (n = 137)				20.6** (5.43)		10.0; 31.3			0.78 (2.62)		-4.36; 5.92			32.2** (8.21)		16.1; 48.3			51.7** (11.3)	29.5; 73.9		252.8 (241.2)	-220.0; 725.7		
AH_NOTADH_+IA (n = 43)				14.1* (6.54)		1.33; 26.9			4.71 (3.90)		-2.92; 12.3			13.3 (8.85)		-3.98; 30.7			29.1* (13.2)	3.18; 55.0		142.9 (265.9)	-378.3; 664.3		
AH_ADH_+IA (n = 16)				24.1* (11.3)		1.90; 46.3			-1.18 (3.35)		-7.76; 5.39			56.6** (28.0)		1.65; 111.5			71.6** (26.7)	19.2; 123.9		265.5 (378.4)	-476.0; 1007.2		
AH_ADH_+ACTIVE (n = 8)				12.5 (11.9)		1.90; 46.3			-5.27* (2.48)		-10.1; -0.39			-4.55 (7.99)		-20.2; 11.1			0.20 (16.2)	-31.7; 31.1		-51.3 (357.8)	-75.7; 649.9		
**Confounding variables**																									
Gender (female)				11.6** (4.25)		3.27; 19.9			1.55 (1.47)		-1.34; 4.45			-5.90 (6.93)		-19.5; 7.69			10.8 (8.50)	-5.82; 27.5		-167.4 (132.1)	-426.4; 91.6		
Age				0.26 (0.24)		-0.22; 0.75			0.10 (0.09)		-0.07; 0.28			0.38 (0.44)		-0.48; 1.25			0.63 (0.52)	-0.39; 1.66		1.38 (5.22)	-14.7; 17.5		
WC				-0.01 (0.13)		-0.27; 0.24			-0.04 (0.04)		-0.13; -0.05			0.15 (0.28)		-0.41; 0.71			-0.05 (0.30)	-0.54; -0.65		-6.19 (4.93)	-15.8; 3.47		
Sum of diseases				12.3* (5.34)		1.87; 22.8			1.20 (1.78)		-2.29; 4.71			26.1** (10.1)		6.25; 45.9			37.5** (11.4)	15.0; 60.0		126.0 (170.7)	-208.6; 460.6		

Test GLM. * <5%. ** <1%. S.E. = std. error. Estim.= estimate. WC = waist circumference. CI = confidence interval. sec.= secondary; terc.= tertiary. NOR + ACTIVE = normotensive + physically active. NOR + IA = normotensive + insufficiently active. AH_NOTADH_+ACTIVE = hypertensive not/partially adherent + physically active. AH_NOTADH_+IA = hypertensive not/partially adherent + insufficiently active. AH_ADH_+ACTIVE = hypertensive adherent + physically active. AH_ADH_+IA = active hypertensive adherent + insufficiently active.

### Other variables and measurements

The patient’s weight, height, body mass index (BMI), and waist circumference (WC) were measured according to the protocol of Lohman [24]. Also, cardiovascular diseases reported by the patients (high cholesterol, diabetes, myocardial infarction, and atherosclerosis) were summed and categorised into two groups: patients with two or fewer diseases were assigned as "0", and patients who had three or more conditions were designated as "1".

Gender, age, WC, and the sum of diseases were considered confounding variables.

### Statistical analysis

Descriptive results are presented in proportions, medians and interquartile ranges (IQR). The General Linear Model (GLM), GAMMA family, and link log, which turns the variables into a log but keeps the residues and natural values ​​in their measurement units, were used to examine the interrelationship between AH, treatment adherence, physical activity and health-related costs. After performing the GLM, the marginal effect analysis was performed, which interpreted the log values, estimating the log values ​​in US$ (American dollar). Statistical significance was set at p < 5%. The software used for this analysis was Stata/MP4 16.

## Results

The sample consisted of 306 patients with an average age of 54.3 years (IQR = 53.4; 55.3), 52.1% (n = 160) were men, and 66.4% were hypertensive. The AH_ADH_+ACTIVE group presented higher costs with consultations (US$ 42.0 [IR = 32.5]) than the NOR + ACTIVE and NOR + IA. However, in the medication costs, their expenditures (US$ 11.4 [IR = 19.5]) were lower than AH_NOTADH_+ACTIVE (US$ 43.4 [IR = 64.7]), and AH_ADH_+IA (68.1 [54.2]) patients. See Table 1.

The AH + IA group were significantly associated with higher health care costs for consultation US$ 23.5 (95%CI = 14.3; 32.8), medications US$ 43.0 (95%CI = 28.4; 57.5) and total primary care US$ 67.0 (95%CI = 48.1; 85.9) in a crude analyse (Table 2).

When confounders were adjusted, this significant association remained for consultations US$ 20.00 (95%CI = 9.90; 30.2), medications US$ 34.20 (95%CI = 18.9; 49.5) and total primary care US$ 51.60 (95%CI = 30.2; 72.9) when compared the AH + IA group to the NOR + ACTIVE. About gender, female sex was related to higher consultation healthcare cost (US$ 11.6 [95%CI = 3.36;19.8]). Furthermore, the patients who had three or more illnesses had higher costs in consultations US$ 12.70 (95%CI = 2.37; 23.1), medication US$ 25.80 (95%CI = 6.17; 45.6) and total primary care US$ 38.10 (95%CI = 15.10; 60.5) than those with two or fewer conditions. See Table 3.

In the crude model (Table 4), those on groups AH_NOTADH_+ACTIVE, AH_NOTADH_+IA and AH_ADH_+IA, had higher consultation, medications, and total primary health care costs than those that NOR + ACTIVE. In the adjusted model (Table 5), the patients with three or more diseases had higher expenditures for primary care costs (consultations US$ 12.30, medication US$ 26.10 and total primary care US$ 37.50) than those with two or fewer diseases. Moreover, in the case of cost attributed to consultation, women spent significantly more than men (US$ 11.60 [95%CI = 3.27;19.9]).

## Discussion

To our best of knowledge, this is the first study exploring the interrelationships between HA, primary care and hospital costs, adherence to treatment and physical activity and through the analysis of patients’ medical records in a city in the interior of São Paulo - Brazil. The main findings showed that, although the physically active, treatment adherent AH patients have higher costs in consultations (US$ 42.0 [IR = 32.5]) and the costs of their medicines (US$ 11.4 [IR = 19.5]) are lower than HA_NOTADH_+ACTIVE, and AH_ADH_+IA patients. Thus, treatment adherence coupled with physical activity can be a robust tool in reducing drug costs. In our study, the non-adherent groups with AH, regardless of their usual physical activity, had higher BMI, weight, WC, hospital costs, and more comorbidities ​​than the physically active + normotensive group (reference). Corroborating our findings, a study that verified the effect of six months of functional exercises and walking on AH showed that patients with AH had higher BMI, WC, systolic (SBP) and diastolic blood (DBP) pressure than the normotensive group [25]. Elevated values ​​may be associated with the presence of AH since the disease itself is a risk factor for high BMI, WC, and other cardiovascular diseases [6].

Female patients showed US$ 11.60 more than men in consultation costs. This cost difference can be explained by women’s specific needs due to their biological specificity, leading to the need for more periodic visits to doctors throughout their lives. For example, of the 2,500,000 women reporting gynaecological disorders in the USA, those aged 45 and ≥ 65 years old had higher medical costs due to menopausal symptoms [26]. Another explanation that may contribute to our findings is that policies aimed at men’s health are recent and do not yet have gender equity in seeking health services. Also, there is still a preconception coming from men for the search for consultations, thus resulting in less demand [27].

Patients with three or more diseases had higher costs of health services. This observation could be because the more disease an individual has, the greater the need for consultations, medications, treatments, and the greater the complexity of their health status, the greater their health services costs [28, 29]. Unfortunately, few studies in the literature examine the impact of three or morbidities on the health service costs of individuals.

The increased body composition in the hypertensive population can be explained by the fact that AH worsens total and abdominal obesity, contributing to the increase in blood pressure, which explains the high values ​​of SBP and DBP [30]. The higher health care costs associated with AH can be explained by the fact that it is a chronic disease that requires continuous treatment. Treatment adherence is crucial in controlling blood pressure. It contributes to the reduction of complications [31, 32], leading to a 30 to 45% reduction in stroke cases and 15% in the incidence of myocardial infarction [33] and overall health costs.

The AH + ACTIVE group showed a significant difference only for consultation services. In contrast, the group that adhered to the treatment and were physically active showed a significant difference only for the costs of laboratory tests. Evidence shows that physically active AH patients have about US$ 50 reduced health care costs than physically inactive AH patients [15]. Also, data from another study showed that treatment adherence lower health costs (US$ 7 182) than treatment non-adherence (US$ 7 560) [32]. Furthermore, a study including 806 individuals, 95.9% hypertensive (28.6% were physically active) and 62.5% diabetic (26.7% physically active), identified that physical activity reduces medication costs by 395% [14]. Also, the literature indicates that an increase in physical activity membership by 70% could result in 332 million euros health savings in five European countries [8]. In all, evidence suggests that treatment adherence and habitual physical activity, together and in isolation, minimises health costs in patients with AH.

Summarily, physical activity can potentially reduce costs with medicines and other health-related costs. However, comparing primary care costs to secondary/tertiary care costs for patients with AH, the former is usually higher than the latter because of fewer complexities associated with secondary/tertiary care.

This study was limited in measuring the number of times per day or week that the hypertensive patient defaulted treatment. Thus, it couldn’t quantify treatment adherence in days, weeks or months, a point that may be important in future studies. Another limitation is the lack of evaluation of the main reasons for treatment non-adherence. Nonetheless, our study’s robust methodology offered significant results for public health policies to improve individuals’ health finances and its resulting quality of life. It also presents critical findings to guide clinicians and health managers in educating AH patients on the importance of treatment adherent and physical activity in their overall health outcomes.

## Conclusion

Although been adherent to the treatment presented relationship with higher healthcare costs, physical activity where related to lower costs. The combination of both, to be adherent and physical active, did not presented difference on health care costs when compared to be normotensive and physical activity participants showing the importance of the combination of these two important variables in the hypertension treatment.

## Data Availability

The datasets used and/or analysed during the current study available from the corresponding author on reasonable request.

## List of Abbreviations

AH: Arterial hypertension
ICD-10: International Classification of Diseases
HPA: Habitual Physical Activity
NOR + ACTIVE: Normotensive + physically active
AH + ACTIVE: Hypertensive + physically active
AH + IA: Hypertensive + insufficiently active
NOR + IA: Normotensive + insufficiently active
AH_NOTADH_+ACTIVE: Hypertensive not/partially adherent + physically active
AHN_OTADH_+IA: Hypertensive not/partially adherent + insufficiently active
AH_ADH_+ACTIVE: Hypertensive adherent + physically active
AH_ADH_+IA: Active hypertensive adherent + insufficiently active
WC: Waist circumference
IQR: Interquartile ranges
GLM: General Linear Model
US$: American dollar
CI: Confidence interval
IR: Interquartile range
